# Pneumatic Retinopexy: An Experience of 12 Years at a Tertiary Care Hospital

**DOI:** 10.7759/cureus.46180

**Published:** 2023-09-29

**Authors:** Mohammad Owais Arshad, Khawaja Muhammad Ammar Ali Javed, Anum Javed, Muhammad Hanif Chatni, Usman Vayani

**Affiliations:** 1 Ophthalmology, Patel Hospital, Karachi, PAK; 2 Ophthalmology, University College London, London, GBR

**Keywords:** single-operation success, vitreoretinal surgery, visual outcomes, retinal detachment, pneumatic retinopexy

## Abstract

Purpose

The purpose of this study is to retrospectively analyze single-operation success (SOS) rates of pneumatic retinopexy (PR) for the treatment of rhegmatogenous retinal detachment (RRD) and to identify the predictors of treatment outcomes.

Methods

Sixty-one eyes of 61 patients who underwent PR for RRD during a period of 12 years were included in this study. Patient demographics along with pre-treatment clinical characteristics were recorded. Treatment outcomes in terms of best-corrected visual acuity (BCVA) and anatomical characteristics were reported including any post-operative complications. Visual outcomes were compared according to pre-treatment characteristics and between groups of the eyes achieving SOS and those requiring additional surgery.

Results

SOS was achieved in 37 (61%) eyes. Twenty-four (39%) eyes required one (36%) or two (3%) additional procedures. There was no significant association between pre-treatment characteristics and treatment failure. SOS eyes had significantly better visual outcomes (P=0.002), and so did those with macula-on status (P=0.003). New/missed breaks (9.8%) and proliferative vitreoretinopathy (PVR) (4.9%) were the most common causes of failure.

Conclusions

We found PR to be a beneficial technique for the treatment of RRD in this cohort. PR remains the least invasive treatment modality, and as a cost-effective technique, it is especially useful in low/middle-income countries such as Pakistan.

## Introduction

Pneumatic retinopexy (PR) is a minimally invasive technique used for the repair of rhegmatogenous retinal detachment (RRD). This technique was originally described by Hilton and Grizzard [1] in 1986 and has since developed into a well-accepted and useful tool in retinal surgery [2]. In the past, the eligibility criteria for considering PR included findings comprising a single break or the presence of multiple breaks occupying a single clock hour with their location being in the superior eight clock hours of the retina.

The identification of all retinal breaks with certainty is dependent on the prerequisite that the media is adequately clear. Furthermore, it is essential to ascertain the ability of the patient to position suitably for gas tamponade of these breaks [3,4].

The eyes that are found to have opacities in the media, the presence of proliferative vitreoretinopathy (PVR), or breaks located in the inferior portion of the retina are generally excluded from the surgical criteria and are more likely to benefit from other procedures. It is however worth noting that there have been reports of success with PR in conditions that do not fit into these strict exclusion criteria such as greater than one clock hour separation of breaks, limited PVR [5], inferior location of breaks [6-8], findings of giant retinal tears [9], mild vitreous hemorrhage, or media opacities [5,10,11]. However, the corresponding anatomical success rates in these studies were lower compared to those observed when the classic exclusion criteria were adhered to.

The anatomical success rates for PR that have been reported previously range between 43.7% and 93.5% and are associated with complication rates similar to those associated with scleral buckling (SB) [1-5]. Even though PR is generally considered inferior to pars plana vitrectomy (PPV) and SB in terms of success rates, it presents an opportunity to steer past a major operating room procedure and hence general anesthesia, increased financial burden, and associated surgical risks. PR is a cost-effective and time-efficient technique, and it has been shown to be not inferior to other procedures as far as final visual outcomes are concerned even when there is an indication for additional surgeries.

This study aims to explore the visual and anatomical outcomes for patients undergoing PR for the repair of RRD at the ophthalmology department in a tertiary care hospital in Karachi, Pakistan.

## Materials and methods

This is a consecutive, non-comparative, retrospective case series conducted in the ophthalmology department of Patel Hospital, Karachi, Pakistan. The study period was six months after the approval of the Ethical Review Committee (ERC). Non-probability purposive convenience sampling was used.

The medical records of all patients requiring PR for the management of primary RRD were accessed between January 1, 2010, and December 31, 2021. Patient files were assessed to collect patient demographics, clinical findings on examination, additional surgeries, and associated complications.

The data obtained included patient age, sex, laterality of affected eye, the time elapsed since symptom onset, the number of retinal breaks, location of retinal breaks including clock hours of RRD, findings of vitreous hemorrhage, macular status, lattice degeneration, retinal adhesive modality, the type of gas applied for PR, phakic status, pre- and post-operative visual acuity, complications encountered, follow-up times, and the use of additional procedures if required.

All patients over the age of 18 years treated with PR for the management of primary RRD with a single clock hour break between eight clock hours and four clock hours of the retina, no PVR or PVR A, no significant media opacity, and no history of glaucoma were included in this study. The exclusion criteria included age under 18 years, previous posterior segment surgery, loss to follow-up after two weeks, or a history of other ocular diseases such as endophthalmitis, uveitis, or neovascular age-related macular degeneration.

PR was performed by two consultant retinal surgeons at the department as per standard procedure protocol [12]. However, a standardized protocol was not used in the selection of patients undergoing the procedure; rather, the decision was made by the individual surgeon treating the patient. The choice of using cryotherapy and/or laser retinopexy and gas (octafluoropropane {C_3_F_8_} or sulfur hexafluoride {SF_6_}) for the tamponade of breaks was at the discretion of the operating surgeon.

The best-corrected visual acuity (BCVA) was measured at presentation and last follow-up using Snellen charts. In the case of the failure of PR, patients underwent additional surgery as a secondary procedure for RRD, which was SB and/or PPV. Retinal reattachment at six-month follow-up or the most recent follow-up if <6 months without the use of additional surgical procedures (PPV/SB) was defined as single-operation success (SOS). The subsequential finding of a new retinal break or that of RRD that presented ≥6 months after PR was not reported as procedure failure but recorded as an unrelated independent occurrence.

Data analysis

All statistical analyses were performed by using Statistical Package for Social Sciences (SPSS) version 21 (IBM SPSS Statistics, Armonk, NY). Descriptive statistics were used to summarize the data. Demographic variables were summarized in terms of mean±standard deviation (SD) for quantitative variables. Percentages and frequencies were used to summarize qualitative variables. Two-sided Student’s t-test or Kolmogorov-Smirnov test was used for the comparison of the success and failure of PR in the case of continuous variables. For categorical variables, the chi-square test or Fisher’s exact test was used. Statistical analyses yielding a P-value of ≤0.05 were considered as statistically significant.

## Results

Patient demographics

A total of 61 eyes of 61 patients were included in this study. Patient demographics are summarized in Table 1. The mean age at presentation was 52±14 (mean±SD) years. Out of 61 patients, 42 (69%) were male, and 19 (31%) were female. The most common comorbidity was diabetes mellitus (23%), followed by hypertension (20%); 5% had both, with the minority (5%) having a family history of RRD.

**Table 1:** Patient demographics, comorbidities, and family history. SD, standard deviation; RRD, rhegmatogenous retinal detachment

Demographics		
Age (years)		
Mean	52	
SD	14	
Sex, N (%)		
Male	42 (69)	
Female	19 (31)	
Comorbidity, N (%)		
Diabetes	14 (23)	
Hypertension	12 (20)	
Both	3 (5)	
Family history of RRD, N (%)		3 (5)

Clinical Characteristics

Within the cohort, the most common site of breaks was the superotemporal quadrant (48%), followed by superonasal (36%), horizontal (13%), and inferior (3%) quadrants. The majority of patients presented with a single break (66%), and retinal detachment in the 4-6 clock hours was the most common (47%) finding. Five (8%) patients presented with vitreous hemorrhage and 14 (23%) with an attached macula, whereas 27 (45%) presented with lattice degeneration. Most patients received cryopexy (71%), while the remainder received a combination of laser and cryopexy (29%). Most of the patients received SF_6_ gas for tamponade (97%). Most eyes were classified as phakic (54%), followed by pseudophakic (44%) and aphakic (2%). The majority of patients did not require any additional surgery for RRD (61%). Of those that did, 36% required a single procedure, whereas 3% required two procedures. The mean follow-up period was 12±1.29 (mean±SD) months. Clinical characteristics along with the corresponding SOS rate are presented in Table 2.

**Table 2:** Clinical characteristics of the eyes that underwent PR along with treatment outcomes and results of significance testing. SF_6_, sulfur hexafluoride; C_3_F_8_, octafluoropropane; PR, pneumatic retinopexy

Characteristics	All patients, N (%)	Single-operation success, N (%)	Additional surgery required, N (%)	P-value
Location of breaks				
Superonasal	21 (36)	13 (21)	8 (13)	0.362
Superotemporal	30 (48)	19 (31)	11 (18)	
Horizontal	8 (13)	3 (5)	5 (8)	
Inferior	2 (3)	2 (3)	0	
Number of breaks				
One	40 (66)	26 (43)	14 (23)	0.338
Multiple	21 (34)	11 (18)	10 (16)	
Clock hours of retinal detachment				
0-3	20 (32)	14 (23)	6 (10)	0.508
4-6	29 (47)	17 (28)	12 (19)	
7-12	13 (21)	6 (10)	6 (10)	
Vitreous hemorrhage on presentation	5 (8)	2 (3)	3 (5)	0.324
Lattice degeneration	27 (45)	15 (25)	12 (20)	0.467
Macula attached	14 (23)	11 (18)	3 (5)	0.118
Retinopexy used				
Laser and cryopexy	18 (29)	10 (17)	8 (13)	0.598
Cryopexy	43 (71)	27 (44)	16 (26)	
Gas used				
SF_6_	59 (97)	35 (61)	24 (39)	
C_3_F_8_	2 (3)	0	2 (100)	
Phakic status				
Phakic	33 (54)	21 (34)	12 (20)	0.587
Pseudophakic	27 (44)	12 (24)	12 (20)	
Aphakic	1 (2)	1 (2)	0	
Number of additional surgeries required	N (%)			
1	22 (36)			
2	2 (3)			
None	37 (61)			

Anatomical and Visual Outcomes

SOS was achieved in 37 eyes (61%). Either the remainder 24 eyes (39%) did not have retinal attachment achieved with PR or there was re-detachment post-procedure. When the eyes achieving SOS with PR were compared to those requiring additional surgery for RRD, there was no significant association with better or poorer outcomes with regard to pre-operative characteristics (Table 2). The median BCVA pre-operatively was 20/400 (range: 20/20 to light perception) for all eyes, which subsequently improved to a median BCVA of 20/25 (range: 20/20 to hand motion) upon examination at the last follow-up. A BCVA of ≥20/40 was found in 43 eyes (70.5%). Both the SOS eyes and the ones requiring additional surgery had a median pre-operative BCVA of 20/400. The median BCVA at the last follow-up was 20/25 in the SOS group and 20/33 for the additional surgery group (P=0.002). A BCVA of ≥20/40 was achieved in 49.2% of SOS eyes as compared to 21.3% in those requiring additional surgery (P=0.024). Pre-operative and post-operative visual acuity is displayed in Table 3.

**Table 3:** Pre- and post-operative visual acuity. *Statistically significant. LP, light perception; HM, hand movement; BCVA, best-corrected visual acuity; F/U, follow-up

	Pre-operative BCVA, median (range)			BCVA at the last F/U, median (range)			Number (%) of eyes with ≥20/40 BCVA at the last F/U	Number (%) of eyes with <20/200 BCVA at the last F/U
	Snellen		Decimal	Snellen	Decimal			
All eyes	20/400 (20/20 to LP)		0.05 (1.00 to LP)	20/25 (20/20 to HM)	0.80 (1.00 to HM)		43 (70.5)	7 (11.5)
Macula-on eyes (n=15)	20/29 (20/20 to HM)		0.70 (1.00 to HM)	20/22 (20/20-20/80)	0.90 (1.00-0.25)		14 (23.0)	0
Macula-off eyes (n=46)	20/400 (20/20 to LP)		0.05 (1.00 to LP)	20/29 (20/20 to HM)	0.70 (1.00 to HM)		29 (47.5)	7 (11.5)
P-value	0.005*			0.003*			0.026*	0.108
Single-operation success eyes (n=37)	20/400 (20/20 to LP)	0.05 (1.00 to LP)		20/25 (20/20-20/400)		0.80 (1.00-0.05)	30 (49.2)	1 (1.6)
Eyes requiring additional surgery (n=24)	20/400 (20/20 to LP)	0.05 (1.00 to LP)		20/33 (20/20 to HM)		0.60 (1.00 to HM)	13 (21.3)	6 (9.8)
P-value	0.714			0.002*			0.024*	0.008*

Post-operatively, 17 (27.9%) patients were found to have persistent sub-retinal fluid (SRF), whereas in 44 (72.1%) patients, it resolved. Eight (13.1%) patients were found to have a new or missed break, two (3.3%) had a reopening of the original break, seven (11.5%) had PVR, five (8.2%) had an epiretinal membrane, two (3.3%) had sub-retinal gas, and eight (13.1%) had a subsequent cataract surgery. Post-operative adverse outcomes are presented in Table 4 along with the SOS rate and p-values for significance testing.

**Table 4:** Complications and adverse events among the study cohort. *Statistically significant. PVR: proliferative vitreoretinopathy

	All patients, N (%)	Single-operation success, N (%)	Eyes requiring additional surgery, N (%)	P-value
Sub-retinal fluid (SRF)				
Persistent	17 (27.9)	4 (6.6)	13 (21.3)	<0.0001*
Resolved	44 (72.1)	33 (54.1)	11 (18.0)	
New or missed break	8 (13.1)	2 (3.3)	6 (9.8)	0.027*
Reopening of original break	2 (3.3)	1 (1.6)	1 (1.6)	0.754
PVR	7 (11.5)	4 (6.6)	3 (4.9)	0.840
Epiretinal membrane	5 (8.2)	1 (1.6)	4 (6.6)	0.052
Sub-retinal gas	2 (3.3)	1 (1.6)	1 (1.6)	0.754
Subsequent cataract surgery	8 (13.1)	2 (3.3)	6 (9.8)	0.027*

## Discussion

Over the past two decades, it has been observed that PR is an underutilized technique for the initial management of RRD. This is perhaps due to the fear of retinal breaks reopening post-operatively coupled with a low threshold for requiring additional surgery. However, the benefits of PR in the initial management of RRD when compared to techniques such as SB and PPV are well-known among vitreoretinal surgeons. The findings of this study strengthen this notion observed in this part of the world [13]. The success rate for PR originally reported by Hilton and Grizzard [1] was 90%. However, similar studies conducted subsequently have shown variable success rates ranging from 60% to 82% [7-12]. The findings of this study report a SOS rate of 61%, which is on the lower end of the range reported previously. They are however close to the findings reported by Dhami et al. with a SOS rate of 60% [13]. It is worth mentioning that there is a significant disparity between success rates in different parts of the world where higher success rates are usually reported in the developed world, whereas those reported in Asian and Arabian ethnicities range from 60% to 75%. Our reported SOS rate of 61% lies within this range [2,5,14,15].

It has previously been observed that anatomical and visual success depends on early presentation and the phakic status of the eye. A comprehensive review of 81 studies including 4,138 eyes between the years 1986 and 2007 conducted by Chan et al. reported that the PR of phakic eyes resulted in a higher SOS (71%-84%) as compared to pseudophakic eyes (41%-67%) [16]. Likewise, in this study, the majority of patients’ eyes were found to be phakic (54%) upon clinical examination, and presentation was at a median of 10 days post symptom onset. Several studies have similarly reported a lower success rate of PR in the eyes that are pseudophakic or aphakic when compared to the success of PR in phakic eyes [4,8,16,17]. A study by Davis et al. reported a success rate of 57.1% in pseudophakic eyes compared to 67.5% in phakic eyes [11]. In addition, Rootman et al. found a significant association between pseudophakic status and PR failure in multivariate analysis [18]. Our results showed a SOS rate of 34% in phakic eyes. However, the SOS rate in pseudophakic eyes was 24% (P=0.58), thereby conforming to the findings of the aforementioned studies.

There are various proposed reasons for this observation; it has been noticed that pseudophakic eyes with posterior capsule rupture and intraocular lens placement in the sulcus have poor dilatation and are at an increased risk of anterior movement of gas bubble, thereby making indirect ophthalmoscopy difficult due to limited fundal view. Consequently, this results in the incorrect localization of breaks and missed breaks, which increases the likelihood of PR failure [17]. Moreover, optical aberrations and posterior capsule opacification render the procedure difficult and associate it with higher rates of failure [14].

The most common site for retinal breaks is the superotemporal quadrant. It is also the most frequent site with single breaks and detached retina [19]. Likewise, the majority of the eyes (48%) in this study had superotemporal breaks. Superotemporal breaks result in inferior field obscuration and are likely to cause significant loss of vision. This presentation most probably compels the patient to seek help urgently within the ophthalmology department in the early phases of visual impairment.

It was observed that macula-on eyes had a higher BCVA on their last follow-up rate when compared to macula-off. This is consistent with the findings of Dhami et al. where macula-on eyes were also associated with a higher SOS rate [13]. Furthermore, the location of breaks also seems to have a role to play as superior breaks had better SOS rate when compared to inferior breaks. This observation is supported by the findings of a larger study by Tornambe, which suggested that a SOS rate of 97% is achievable given that the eyes are phakic and have RRD in only one quadrant, the presence of only a single break in the upper two-thirds of the fundus, and the use of post-operative 360 degrees of prophylactic laser [5].

Post-operatively, 17 (27.9%) patients had persistent SRF. In contrast to conventional SB, SRF is generally not drained as part of the PR procedure. This in turn leads to the slow reabsorption of SRF. It is believed that the slow reabsorption of SRF permits a slow settling of the outer part of the retina, which renders effective photoreceptor readjustment against the retinal pigment epithelium layer leading to an improved visual outcome and hence the rescue of visual impairment [20].

PR failure is almost always observed within the early post-operative window of the first month [20]. This has been observed previously and is most likely associated with poor patient compliance with regard to positioning. It is therefore suggested that decisions around performing PR should include pre-operative discussions with the patient and assessment in order to ascertain whether they are able to comply with the prescribed positioning and are willing to adhere to strict follow-up [21].

One of the key benefits of PR versus other procedures is the significantly less tissue damage and the improved regain of vision [22]. PR has also been shown to cause a lesser myopic shift when compared to SB [23]. Additionally, it has been reported to result in an enhanced regain of vision when compared to PPV [24].

Limitations

There are several limitations to this study including its retrospective design and a SOS rate lower than that observed in the developed world. There may be various reasons for this lower success rate, but new/missed breaks and the progression of PVR were perhaps the most likely reasons for the failure of PR in this study. In addition, there is a selection bias among surgeons in selecting cases suitable for PR, and it has been observed that young retinal surgeons prefer PPV as the procedure of choice for RRD [25]. This can be attributed to the ease of availability of minimally invasive vitreoretinal surgery that is PPV (25G and 27G) or the fear of PVR formation. Moreover, there was no standardized protocol for the selection of patients suitable for the procedure in this study, and the choice of gas used for the tamponade of breaks was at the discretion of the individual surgeon performing the procedure. These selection biases are likely to result in poorer success rates. Furthermore, our study cohort did not undergo strict measures in monitoring position post-procedure, which could also be a factor contributing to the lower SOS rate observed. PR, however, remains a useful and effective technique within the options available to vitreoretinal surgeons in treating RRD, being perhaps the least invasive method of treating RRD with reduced post-operative morbidity along with its cost-effectiveness. The cost-effectiveness of this technique is of particular importance here in a developing country where funding is scarce and is coupled with an increased incidence of RRD among the adult population. Thus, PR provides a useful solution to overcome this situation.

## Conclusions

In conclusion, the findings of this study support the application of PR for the treatment of primary RRD. PR remains a useful tool in the treatment of RRD with several benefits including but not exhaustive of being the least invasive method for the treatment of RRD, being time-efficient and cost-effective, and having a prolific safety profile. Several factors, however, need to be considered to make the usage of this useful technique more widespread here in Pakistan, which includes the careful selection of eligible patients. Finally, it may be inferred that by opting for PR, effective vision recovery can be achieved in a cost-effective way, thereby reducing the financial burden on an already underfunded healthcare system and on patients belonging to lower social classes within this society.
